# Supplementary material for: Clinicopathological Appearance of Epidermal Growth-Factor-Containing Fibulin-like Extracellular Matrix Protein 1 Deposition in the Lower Gastrointestinal Tract: An Autopsy-Based Study
Source: Int J Mol Sci. 2024 Jul 10;25(14):7581. doi: 10.3390/ijms25147581 (PMC11277079; doi:10.3390/ijms25147581)
Supplement: Supplementary file 1 [file ijms-25-07581-s001.zip › ijms-3080531-supplementary.pdf]

ONLINE SUPPLEMENTAL MATERIAL

Clinicopathological appearance of epidermal growth factor-containing fibulin-like extracellular matrix protein 1 deposition: An autopsy-based study

Shojiro Ichimata[1], Yukiko Hata[1], Koji Yoshida[1], Naoki Nishida[1]

[1] Department of Legal Medicine, Faculty of Medicine, University of Toyama, Toyama, Japan

List of Supplemental material

Supplemental Table: 3

Supplementary Table S1. All clinical and pathological information of the cases.

#	Age	Sex	Type of death	Cause of death	Const*	LBD**	Thyroiditis	ATTR-CA	S-SI	S-Colon
1	88	M	Accident	Asphyxia	None	Negative	Negative	Negative	A	A
2	81	M	Accident	Drowning	None	1	Negative	Positive	A	A
3	80	M	Illness	ACI	None	Negative	Negative	Negative	A	A
4	83	M	Accident	Trauma	None	Negative	Negative	Positive	A	A
5	82	M	Illness	SAH	Unknown	Negative	Negative	Negative	NA	A
6	94	M	Accident	Drowning	None	Negative	Negative	Positive	A	A
7	89	F	Suicide	ADP	None	Negative	Negative	Negative	NA	A
8	100	F	Illness	Lund cancer	Unknown	3	Negative	Negative	A	A
9	87	M	Accident	Trauma	Positive	Negative	Negative	Negative	A	A
10	90	M	Accident	Hypothermia	None	Negative	Negative	Negative	A	A
11	85	F	Accident	Trauma	Possible	5	Positive	Negative	A	A
12	83	M	Accident	Drowning	None	4	Negative	Negative	NA	A
13	88	F	Accident	Drowning	Possible	Negative	Negative	Negative	A	A
14	90	F	Accident	Burn	None	5	Negative	Negative	NA	A
15	84	F	Accident	CMP	Possible	Negative	Negative	Negative	A	A
16	80	F	Suicide	Drowning	Unknown	Negative	Negative	Negative	A	A
17	83	M	Suicide	Drowning	None	Negative	Negative	Positive	A	A
18	85	F	Illness	ACI	None	Negative	Negative	Negative	A	A
19	82	F	Homicide	Asphyxia	None	Negative	Negative	Negative	NA	A
20	88	M	Suicide	Drowning	Definite	Negative	Negative	Negative	A	A
21	94	F	Accident	SAH	None	Negative	Negative	Negative	A	A
22	83	M	Accident	ATE	None	Negative	Negative	Negative	NA	A
23	97	F	Accident	Heat stroke	None	3	Negative	Positive	NA	A
24	86	F	Accident	Heat stroke	None	Negative	Positive	Negative	NA	A
25	90	F	Accident	Drowning	Definite	Negative	Negative	Positive	NA	A
26	80	F	Suicide	Drowning	None	Negative	Negative	Negative	NA	A
27	80	M	Suicide	Asphyxia	None	Negative	Negative	Negative	NA	A
28	82	F	Accident	Hypothermia	None	Negative	Negative	Negative	NA	A
29	88	M	Illness	ACF	None	Negative	Negative	Negative	NA	A
30	86	F	Accident	Hypothermia	None	4	Negative	Negative	A	A
31	81	M	Accident	Trauma	None	1	Negative	Negative	A	A
32	87	M	Illness	ACF	Possible	Negative	Negative	Negative	NA	A
33	80	M	Suicide	Asphyxia	Unknown	Positive	Negative	Negative	NA	A
34	81	F	Illness	Infection	Unknown	Negative	Negative	Negative	A	A
35	91	M	Accident	Drowning	None	3	Negative	Negative	A	A
36	85	F	Illness	CCF	Unknown	Negative	Positive	Negative	A	A
37	84	F	Accident	SAH	None	Negative	Negative	Negative	NA	A
38	84	F	Illness	Peritonitis	None	1	Negative	Negative	A	A
39	89	M	Accident	Drowning	None	Negative	Negative	Positive	NA	A
40	86	M	Accident	Hypothermia	None	Negative	Negative	Negative	NA	A

Abbreviations: A, available; ACI, acute cardiac failure; ACF, acute cardiac failure; ATE, acute thromboembolism; ADP, acute drug poisoning; CCF, congestive cardiac failure; CMP, carbon monoxide poisoning; F, female; LBD, Lewy body disease; M, male; NA, not available; SAH, subdural hemorrhage; S-Colon, specimens from the colon; S-SI, specimens from the small intestine. * Definite refers to cases with a clinical diagnosis of constipation. Possible refers to cases with no clinical diagnosis but with laxative medications or pseudomelanosis coli. ** Evaluated based on the Braak's LBD grading [1].

Supplementary Table S2. All pathological results of the small intestine evaluation.

#	SI#	MM			SM (V)			MP			AP			SS (V)			SS (I)			S			TS	Group*	AGBR	EF	ATTR
		EM	CR	IHC	EM	CR	IHC	EM	CR	IHC	EM	CR	IHC	EM	CR	IHC	EM	CR	IHC	EM	CR	IHC					
1	1	0	0	0	1	1	2	0	0	1	0	0	1	1	2	2	0	0	2	1	2	3	19	Intermediate	Positive	Negative	Negative
2	2	0	0	0	3	3	3	1	1	2	2	2	3	3	3	3	2	2	3	3	3	3	45	Severe	Positive	Negative	Negative
3	3	0	0	0	0	0	1	0	0	0	0	0	0	0	1	1	1	1	2	1	1	2	11	Mild	Negative	Negative	Negative
4	4	0	0	0	0	0	2	0	0	0	0	0	0	0	0	1	0	0	1	0	0	2	6	Mild	Negative	Negative	Positive
6	5	0	0	0	1	1	3	0	0	1	0	0	2	3	3	3	2	2	3	2	2	3	31	Severe	Positive	Negative	Positive
8	6	0	0	0	0	0	1	0	1	2	1	2	3	1	2	3	0	0	2	0	0	1	19	Intermediate	Positive	Positive	Positive
9	7	0	0	0	0	0	2	0	0	1	0	0	0	0	2	3	0	1	3	1	1	3	17	Intermediate	Positive	Negative	Negative
10	8	0	0	0	0	1	2	1	1	2	0	0	1	1	2	2	1	1	3	2	2	3	25	Severe	Positive	Negative	Negative
11	9	0	0	0	0	0	1	0	0	0	0	0	0	0	0	1	0	0	1	0	0	2	5	Mild	Negative	Negative	Negative
13	10	0	0	1	0	1	3	1	1	2	0	0	1	1	2	3	1	2	3	2	2	3	29	Severe	Positive	Negative	Negative
15	11	0	0	0	0	0	2	0	1	2	0	0	1	1	2	3	1	2	3	2	2	3	25	Severe	Positive	Negative	Negative
16	12	0	0	0	0	0	2	0	0	1	0	0	0	0	1	2	0	0	2	0	1	2	11	Mild	Negative	Negative	Negative
17	13	0	0	1	0	0	2	0	0	1	0	0	0	0	0	2	0	0	2	1	1	3	13	Mild	Negative	Negative	Positive
18	14	0	0	0	0	0	1	0	0	0	0	0	0	0	0	1	0	0	1	0	0	1	4	Mild	Negative	Negative	Negative
20	15	0	0	0	0	0	2	0	0	2	0	0	1	0	1	3	0	1	2	2	2	3	19	Mild	Negative	Negative	Negative
21	16	0	0	0	0	0	1	0	0	0	0	0	0	1	2	2	0	0	2	0	1	3	12	Intermediate	Positive	Negative	Negative
30	17	0	0	1	0	0	2	0	1	2	0	0	2	1	2	2	1	1	1	2	2	3	23	Severe	Positive	Negative	Negative
31	18	0	0	0	0	0	1	0	0	2	0	0	1	0	1	2	0	0	2	0	0	2	11	Mild	Negative	Negative	Negative
34	19	0	0	0	0	0	1	0	0	0	0	0	0	1	1	2	0	1	3	1	1	3	14	Intermediate	Positive	Negative	Negative
35	20	0	0	0	0	0	1	0	0	1	0	0	0	2	2	3	1	1	2	1	2	2	18	Intermediate	Positive	Negative	Negative
36	21	0	0	0	0	0	1	0	0	1	0	0	1	0	0	2	0	0	2	0	1	3	11	Mild	Negative	Negative	Negative
38	22	0	0	0	0	0	1	0	0	1	0	0	0	0	0	1	0	0	1	0	0	1	5	Mild	Negative	Negative	Negative

Abbreviations: AGBR, apple-green birefringence; ATTR, amyloid transthyretin; CR, Congo red grading; EF, elastofibrosis; EM, elastica-Masson grading; I, interstitium; IHC, immunohistochemistry for EFEMP1; MM, mucosa and muscularis mucosa; MP, muscularis propria; S, serosa; SI#, small intestine #; SM, submucosa; SS, subserosa; TS, total score; V, vessel. * Mild (low-TS (<20) and AGBR-negative); Intermediate (low-TS but AGBP-positive); Severe (high-TS and AGBR-positive).

Supplementary Table S3. All pathological results of the colon evaluation.

#	MM			SM (V)			MP			AP			SS (V)			SS (I)			S			TS	Group*	AGBR	EF	ATTR
	EM	CR	IHC	EM	CR	IHC	EM	CR	IHC	EM	CR	IHC	EM	CR	IHC	EM	CR	IHC	EM	CR	IHC					
1	0	0	0	0	0	1	1	0	2	0	0	2	1	2	3	0	0	2	0	0	1	15	Severe	Positive	Negative	Negative
2	0	0	0	3	3	3	3	2	3	2	2	3	3	3	3	1	1	2	0	0	2	39	Intermediate	Negative	Negative	Negative
3	0	0	0	0	0	1	0	0	1	0	0	0	0	0	2	0	0	2	0	0	2	8	Mild	Negative	Negative	Negative
4	0	0	0	0	0	1	0	0	1	0	0	1	0	0	2	0	0	1	0	0	1	7	Mild	Negative	Negative	Positive
5	0	0	1	3	3	3	1	1	2	0	0	2	2	2	3	0	1	2	0	0	1	27	Severe	Positive	Negative	Negative
6	0	0	0	0	1	1	1	0	1	1	1	1	2	2	2	0	0	0	0	0	1	14	Intermediate	Negative	Negative	Positive
7	0	0	0	0	0	1	0	0	1	0	0	1	1	2	3	0	0	2	0	0	1	12	Severe	Positive	Negative	Negative
8	0	0	0	0	0	1	0	1	2	0	0	2	1	2	3	0	0	2	0	0	1	15	Intermediate	Negative	Positive	Negative
9	0	0	1	0	0	2	0	0	1	0	0	1	1	2	3	0	0	2	1	1	3	18	Severe	Positive	Positive	Negative
10	0	0	0	0	0	0	1	1	2	1	1	2	2	2	3	0	1	2	1	1	1	21	Severe	Positive	Negative	Negative
11	0	0	0	0	0	1	0	0	0	0	0	0	0	0	1	0	0	0	0	0	0	2	Mild	Negative	Negative	Negative
12	0	0	0	0	0	0	0	0	0	0	0	0	0	0	1	0	0	1	0	0	1	3	Mild	Negative	Negative	Negative
13	0	0	1	0	0	1	0	0	1	0	0	1	1	1	2	0	0	1	0	0	1	10	Intermediate	Positive	Negative	Negative
14	0	0	1	0	0	1	0	0	2	0	0	1	0	1	2	0	0	1	0	0	1	10	Intermediate	Positive	Negative	Negative
15	0	0	1	0	0	2	0	0	1	0	0	1	0	1	2	0	0	0	0	0	1	9	Mild	Negative	Negative	Negative
16	0	0	0	0	0	1	0	0	0	0	0	0	0	0	1	0	0	0	0	0	0	2	Mild	Negative	Negative	Negative
17	0	0	1	0	0	1	0	0	1	0	0	1	0	0	2	0	0	0	0	0	0	6	Mild	Negative	Negative	Positive
18	0	0	1	0	0	1	0	0	0	0	0	1	0	1	2	0	0	1	0	0	1	8	Mild	Negative	Negative	Negative
19	0	0	0	0	0	0	0	0	0	0	0	0	0	0	1	0	0	0	0	0	0	1	Mild	Negative	Negative	Negative
20	0	0	1	0	0	1	0	0	1	0	0	1	1	2	3	1	1	2	1	1	2	18	Severe	Positive	Positive	Negative
21	0	0	0	0	0	1	0	0	1	0	0	1	0	2	2	0	0	1	0	0	1	9	Mild	Negative	Negative	Negative
22	0	0	2	0	0	2	0	0	1	0	0	1	0	0	1	0	0	0	0	0	0	7	Mild	Negative	Negative	Negative
23	0	0	1	2	2	3	1	1	2	1	2	3	1	2	3	2	2	3	0	1	1	33	Severe	Positive	Negative	Positive
24	0	0	0	0	0	1	0	0	1	0	0	1	1	1	2	0	0	1	0	0	1	9	Mild	Negative	Negative	Negative
25	0	0	0	0	0	1	0	0	0	0	0	0	0	0	1	0	0	0	0	0	1	3	Mild	Negative	Negative	Negative
26	0	0	0	0	0	2	0	1	2	1	1	2	2	2	3	0	0	2	0	0	0	18	Intermediate	Negative	Negative	Negative
27	0	0	0	0	0	1	0	0	1	0	0	1	0	0	1	0	0	1	0	0	0	5	Mild	Negative	Negative	Negative
28	0	0	1	0	0	2	0	1	3	1	2	3	0	1	2	0	0	1	0	0	2	19	Intermediate	Negative	Negative	Negative
29	0	0	0	0	0	1	0	0	1	0	0	1	0	0	2	0	0	1	0	0	1	7	Mild	Negative	Negative	Negative
30	0	0	1	0	0	1	0	0	1	0	0	1	0	1	2	1	1	2	0	0	1	12	Intermediate	Negative	Negative	Negative
31	0	0	0	0	0	1	0	0	1	0	0	1	0	0	2	0	0	1	0	0	1	7	Mild	Negative	Positive	Negative
32	0	0	0	0	0	1	1	1	2	2	2	3	0	1	2	0	0	1	0	0	1	17	Intermediate	Negative	Positive	Negative
33	0	0	1	0	0	1	0	1	2	0	0	2	1	2	3	1	1	2	1	1	1	20	Severe	Positive	Positive	Negative
34	0	0	1	0	0	1	0	0	1	0	0	0	0	1	2	0	0	1	0	0	0	7	Mild	Negative	Negative	Negative
35	0	0	0	0	1	2	0	0	1	0	0	1	1	2	3	0	0	2	0	0	1	14	Severe	Positive	Negative	Negative
36	0	0	2	0	0	1	0	0	2	0	0	1	1	2	3	0	0	2	0	0	1	15	Severe	Positive	Negative	Negative
37	0	0	2	0	1	2	1	1	2	1	1	2	2	2	3	0	0	2	0	0	1	23	Severe	Positive	Positive	Negative

38	0	0	1	0	0	1	0	0	1	0	0	1	0	0	1	0	0	1	0	0	0	6	Mild	Negative	Negative	Negative
39	0	0	1	0	0	1	1	1	2	0	0	2	1	2	2	0	0	1	0	0	0	14	Severe	Positive	Negative	Negative
40	0	0	0	0	0	1	0	0	1	0	0	1	0	1	2	0	0	1	0	0	1	8	Mild	Negative	Negative	Negative

Abbreviations: AGBR, apple-green birefringence; ATTR, amyloid transthyretin; CR, Congo red grading; EF, elastofibrosis; EM, elastica-Masson grading; I, interstitium; IHC, immunohistochemistry for EFEMP1; MM, mucosa and muscularis mucosa; MP, muscularis propria; N#, new number; S, serosa; SM, submucosa; SS, subserosa; TS, total score; V, vessel. * Mild (low-TS (<12) and AGBR-negative); Group 2 (low-TS but AGBR-positive, or high-TS (≥12) but AGBR-negative); Group 3 (high-TS and AGBR-positive.

References

1. Hobbs, C.M.; Burch, D.M.; Sobin, L.H. Elastosis and elastofibromatous change in the gastrointestinal tract: A clinicopathologic study of 13 cases and a review of the literature. *Am. J. Clin. Pathol.* **2004**, *122*, 232–237.
